# Detection of Novel US *Neisseria meningitidis* Urethritis Clade Subtypes in Japan

**DOI:** 10.3201/eid2911.231082

**Published:** 2023-11

**Authors:** Hideyuki Takahashi, Masatomo Morita, Mitsuru Yasuda, Yuki Ohama, Yoshitomo Kobori, Munekado Kojima, Ken Shimuta, Yukihiro Akeda, Makoto Ohnishi

**Affiliations:** National Institute of Infectious Diseases, Tokyo, Japan (H. Takahashi, M. Morita, Y. Ohama, K. Shimuta, Y. Akeda, M. Ohnishi);; Sapporo Medical University, Sapporo, Japan (M. Yasuda);; Private Care Clinic Tokyo, Tokyo (Y. Kobori);; Nagoya Urology Hospital, Aichi, Japan (M. Kojima)

**Keywords:** *Neisseria meningitidis*, meningococci, serogroup, invasive meningococcal diseases, urethritis, urethritis clade, whole-genome sequencing, phylogenetic analysis, bacteria, meningitis/encephalitis, sexually transmitted infections, Japan

## Abstract

*Neisseria meningitidis* causes invasive meningococcal diseases and has also been identified as a causative agent of sexually transmitted infections, including urethritis. Unencapsulated sequence type 11 meningococci containing the gonococcal *aniA-norB* locus and belonging to the United States *N. meningitidis* urethritis clade (US_NmUC) are causative agents of urethral infections in the United States, predominantly among men who have sex with men. We identified 2 subtypes of unencapsulated sequence type 11 meningococci in Japan that were phylogenetically close to US_NmUC, designated as the Japan *N. meningitidis* urethritis clade (J_NmUC). The subtypes were characterized by PCR, serologic testing, and whole-genome sequencing. Our study suggests that an ancestor of US_NmUC and J_NmUS urethritis-associated meningococci is disseminated worldwide. Global monitoring of urethritis-associated *N. meningitidis* isolates should be performed to further characterize microbiologic and epidemiologic characteristics of urethritis clade meningococci.

*Neisseria meningitidis* causes invasive meningococcal diseases (IMDs), such as meningitis and septicemia. *N. meningitidis* is classified into 12 defined serogroups; however, most IMDs are associated with the serogroups A, B, C, W, X, and Y (*1*). Serogrouping is critical for IMD control because meningococcal vaccines have serogroup-specific effects (*2*). Whole-genome sequencing (WGS)–based typing, such as high-resolution core genome multilocus sequence typing (MLST), is the most powerful method for analyzing meningococcal isolates. However, standard MLST, which identifies sequence types (STs) of isolates according to the unique allelic profiles of 7 housekeeping genes, is still applied to meningococcal epidemiology studies because the most invasive isolates belong to a limited number of clonal complexes (CCs). For example, ST11 and the locus variants comprising CC11 meningococci are well-known hypervirulent *N. meningitidis* strains that have caused many pandemics (*3*), including IMD outbreaks that predominately occurred among men who have sex with men (MSM) (*4*–*9*).

*N. gonorrhoeae* is also a human pathogen capable of infecting the urethra, cervix, rectum, and oropharynx. Most gonococcal infections manifest clinically as urethritis in men or cervicitis in women, both of which are sexually transmitted infections (STI). Meningococcus and gonococcus are generally regarded as distinct taxa that cause specific diseases; however, recent findings suggest a greater overlap than was originally reported. *N. gonorrhoeae* is rarely identified as a causative agent of systemic infection; *N. meningitidis* has been reported to cause STIs, such as urethritis. An outbreak of meningococcal urethritis predominantly among MSM was reported in multiple cities in the United States (*10*). Causative agents were identified as CC11 *N. meningitidis* isolates with several unique features and classified as US *N. meningitidis* urethritis clade (US_NmUC) (*11*–*14*). The capsular polysaccharide (*cps*) locus in US_NmUC meningococci is disrupted by insertion sequence (IS) 1301 that replaced *ccsA*, *cssB*, and *cssC* genes and part of the *csc* gene causing loss of encapsulation (*12*). That genetic mutation also caused the loss of wild-type lipooligosaccharide sialylation, which appeared to increase mucosal surface adherence (*15*). Moreover, the factor H binding protein (fHbp), which binds to human factor H and inhibits the alternative complement activation pathway in the human immune system (*16*), was highly expressed in US_NmUC *N. meningitidis* isolates and might promote evasion from immune responses in the human urogenital tract (*12*). The most unique feature of US_NmUC meningococci is their acquisition of the *N. gonorrhoeae* denitrification apparatus that comprises gonococcal alleles encoding nitrate reductase AniA and nitric oxide reductase NorB and the intergenic promoter region, which confers survival in the urogenital tract (*12*,*17*).

Most US_NmUC isolates have been recovered from patients with urethritis in the United States. However, 2 US_NmUC meningococci isolates were identified in 2019 in rectal swab samples from MSM in the United Kingdom (*18*), and 19 US_NmUC meningococci were isolated in Vietnam in 2019 and 2020 (*19*). US_NmUC meningococci have not yet been reported in other countries. We report the genomic and phenotypic features of 3 unencapsulated ST11 urethritis-associated *N. meningitidis* strains isolated in Japan that were phylogenetically close to US_NmUC but classified as novel urethritis meningococcus clade subtypes.

## Methods

### *N. meningitidis* Isolates

Although IMDs are legally notifiable diseases in Japan, STIs caused by *N. meningitidis* are not. In Japan, meningococcal isolates from patients with STIs are typically collected as part of the countrywide gonococcal surveillance program (headed by M.Y.). Urethral swab samples from male patients suspected of having urethritis and cervical swab samples from female patients suspected of having cervicitis were sent to Sapporo Medical University from ≈100 clinics across Japan. We isolated strains by selective growth on Thayer-Martin medium and analyzed those isolates by using Biotyper matrix-assisted/laser desorption time-of-flight mass spectrometry (Beckman Coulter, https://www.beckmancoulter.com) and commercially available mass spectrometry profiles to identify species. We collected >1,000 gonococcal isolates annually and isolated ≈10 *N. meningitidis* strains under the gonococcal surveillance program, in which no misidentification of *N. meningitidis* as *N. gonorrhoeae* has occurred. We characterized 3 *N. meningitidis* isolates at the National Institute of Infectious Diseases by using serologic and genetic analyses.

### Typing and Antimicrobial Drug Susceptibility Tests

We performed serogrouping by using PCR (*20*) and slide agglutination tests with meningococcal rabbit antiserum (Remel, http://www.remel.com, or Difco/Becton Dickinson, https://www.bd.com) and a commercial latex agglutination kit (Pastorex Meningitis assay; Bio-Rad Laboratories, https://www.bio-rad.com). We conducted MLST by using the standard method (*21*). We performed antimicrobial drug susceptibility tests by using E-tests (bioMérieux, https://www.biomerieux.com) and Mueller-Hinton agar with 5% sheep blood (Becton Dickinson), which we interpreted according to the Clinical and Laboratory Standards Institute criteria for agar dilution, as previously described (*22*).

### WGS, Genome Assembly, and Phylogenetic Analysis

We extracted genomic DNA by using the MagMAX DNA Multi-Sample Ultra 2.0 Kit, which we then purified by using the KingFisher Duo Prime Purification System and measured concentrations by using a Qubit dsDNA HS assay kit (all from Thermo Fisher Scientific, https://www.thermofisher.com). We prepared genomic libraries for short read sequencing by using the QIAseq FX DNA Library Kit (QIAGEN, https://www.qiagen.com) and sequenced 300-bp paired-end reads on a MiSeq instrument (Illumina, https://www.illumina.com). For long-read sequencing on a MinION sequencer (Oxford Nanopore Technologies, https://nanoporetech.com), we prepared genomic libraries by using a Rapid Barcoding Kit (Oxford Nanopore Technologies) and sequenced them by using an R9.4.1 flow cell. We basecalled raw data by using Guppy 6.5.7 (*23*) and removed adaptors before assembly by using Porechop 0.2.3 (https://github.com/rrwick/Porechop). We generated draft genome sequences for both long and short reads by using Unicycler version 0.5.0 in conservative mode (*24*) and performed annotations of complete genomes and genome assemblies by using the DDBJ Fast Annotation and Submission Tool (https://dfast.ddbj.nig.ac.jp) (*25*). We used draft genome assemblies for PorA and FetA typing and determining the Meningococcal Deduced Vaccine Antigen Reactivity Index through PubMLST (https://www.pubmlst.org). We performed phylogenetic analyses of *N. meningitidis* from urethritis patients by using 26 publicly available genomes and constructed core gene alignments by using Roary version 3.12.0 and the -s and -e–mafft options (*26*), which were subject to SNP-sites version 2.5.1 (*27*) to extract single-nucleotide variants. We constructed the phylogenetic tree by using IQ-TREE version 2.0.3 (http://www.iqtree.org) with 1,000 ultrafast bootstrap replicates and visualized the tree by using iTOL (*28*).

### Repositories

We deposited the short reads sequence data for NIID835, NIID836, and NIID838 in the DDBJ Sequence Read Archive (https://www.ddbj.nig.ac.jp) under accession nos. DRR494404 (NIID835), DRR494405 (NIID836), and DRR494406 (NIID838) and in the PubMLST database under nos. 135430 (NIID835), 135431 (NIID836), and 135432 (NIID838). The annotated complete genome assemblies of NIID835, NIID836, and NIID838 strains are also available in the GenBank, EMBL (https://www.ebi.ac.uk), and DDBJ databases under accession nos. AP028680 (NIID835), AP028681 and AP028682 (NIID836), and AP028683 (NIID838).

## Results

The 3 J_NmUC *N. meningitidis* strains (NIID835, NIID836, and NIID838) were isolated from 3 men with urethritis that developed 4–5 days after contact with commercial sex workers for oral sexual services (Appendix 1 Table). Although *N. meningitidis* strains from patients with urethritis in Japan are typically classified as ST11026, which is also isolated from healthy carriers (*29*), or ST23, which is also isolated from IMD patients and healthy carriers (*30*), we identified all 3 J_NmUC *N. meningitidis* strains as ST11 (Appendix 1 Table). To further characterize the 3 J_NmUC *N. meningitidis* isolates as urethritis clade meningococci, we performed WGS, phylogenetic, and serologic analyses.

### *aniA-norB* Locus

**Figure 1:**
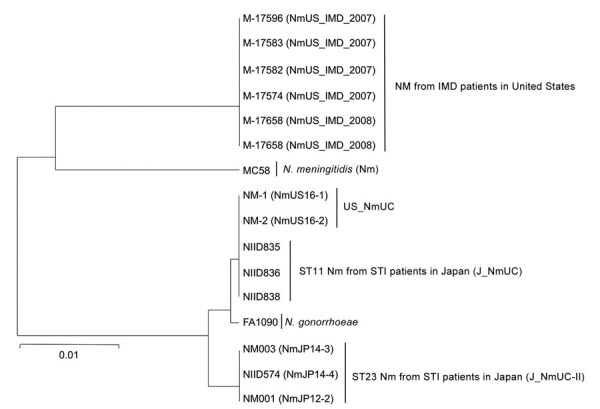
Phylogenetic analysis of the 3.5-kb *aniA-norB* gene locus of *Neisseria* spp. isolates in study detecting novel US *N. meningitidis* urethritis clade subtypes in Japan. Tree was constructed by using the unweighted pair group method with arithmetic mean and 1,000 bootstrap replicates. The gonococcal *aniA-norB* locus was derived from *N. gonorrhoeae* FA1090 (GenBank accession no. NC_002946.2); all others are from *N. meningitidis* isolates. Scale bar indicates nucleotide substitutions per site. IMD, invasive meningococcal disease; Nm, *N. meningitidis*, ST, sequence type; STI, sexually transmitted infection.

We conducted phylogenetic analysis of the 3.5-kb *aniA-norB* gene sequence (Appendix 2 Figure 1) for 3 J_NmUC *N. meningitidis* isolates from Japan, 2 *N. meningitidis* US_NmUC isolates, *N. gonorrhoeae* FA1090 (GenBank accession no. NC_002946.2), *N. meningitidis* MC58 (*31*), and 6 *N. meningitidis* serogroup C isolates from IMD patients in United States that were genetically very close to US_NmUC (*32*) (Figure 1). Moreover, we included 3 ST23 *N. meningitidis* isolates (NM001, NM003, and NIID574) from Japan harboring the gonococcal *aniA-norB* locus (*30*), designated as J_NmUC-II (Figure 1). The *aniA-norB* locus in the 3 ST11 J_NmUC isolates was 100% identical to that in US_NmUC meningococci (*12*,*17*), indicating the *aniA-norB* locus in the 3 J_NmUC strains was of gonococcal origin. In the 3 ST11 J_NmUC and 3 J_NmUC-II isolates, the *aniA-norB* locus was located between *gpxA* and NMB1624 genes (Appendix 2 Figure 1), which was identical to that in US_NmUC *N. meningitidis* strains (*12*). Collectively, those results indicated that the 3 ST11 J_NmUC isolates acquired the gonococcal *aniA-norB* locus, similar to US_NmUC meningococci.

### Serogrouping and *cps* Locus Analysis

**Figure 2:**
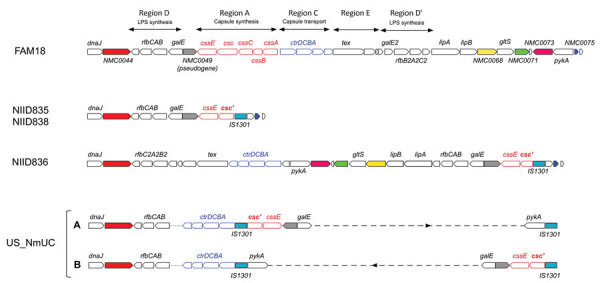
Organization of genes within the *cps* locus of *Neisseria* *meningitidis* isolates in study of detection of novel US *N. meningitidis* urethritis clade subtypes in Japan. *N. meningitidis* isolates from Japan (NIID835, NIID836, NIID838) and United States (US_NmUC) were compared with *N. meningitidis* strain FAM18 (GenBank accession no. AM421808). Open red arrows indicate the *cssA*, *cssB*, *cssC*, *csc*, and *cssE* genes in region A responsible for capsule synthesis and open blue arrows the *ctrD*, *ctrC*, *ctrB*, and *ctrA* genes (in that order) in region C responsible for capsule transport. Insertion sequence IS1301 is indicated. Open reading frames identical to NMC0044 (solid red), NMC0049 (gray), NMC0068 (yellow), NMC0071 (green), NMC0073 (pink), and NMC0075 (blue) in FAM18 are shown for each isolate. Partial deletion is indicated for the *csc* gene (*csc′*). The *cps* locus for US_NmUC had 2 configurations created by a ≈20-kb genome inversion between 2 IS1301 sequences (designated as A and B). Gene alignments in the region between the 2 IS1301 sequences have been omitted and are indicated by the dashed line. Although *ctrD*, *ctrC*, *ctrB*, and *ctrA* genes were shown to be proximal to *dnaJ* (*12*), contigs containing the *dnaJ-rfbC*, *rfbA*, and *rfbB* genes and the *ctrD*, *ctrC*, *ctrB*, and *ctrA* genes (shown on the left side of A and B), as well as 2 IS1301 and *pykA* genes (shown on the right side of A and B), were not connected by our analysis because of the absence of US_NmUC long-read sequences. Therefore, unidentified connections of the 2 contigs are indicated by a dotted line.

Although we initially identified the 3 ST11 J_NmUC *N. meningitidis* strains as serogroup C meningococci (MenC) by PCR (*20*), the strains were agglutination negative when we tested with serogroup C–specific antiserum. To clarify this discrepancy, we characterized the *cps* gene locus (Figure 2). In NIID835 and NIID838 isolates, *cssA*, *cssB*, and *cssC* genes, and part of the *csc* gene (region A) were deleted and replaced with IS1301, but the *ctrABCD* gene cluster (region C) was also deleted. In contrast to 2 copies of IS1301 in US_NmUC isolates (*12*), only 1 copy of IS1301 was found in the *cps* locus of NIID835 and NIID838 isolates. In the NIID836 J_NmUC isolate, deletions of *cssA*, *cssB*, *cssC*, *csc* genes were identical to those in NIID835 and NIID838, but the *ctrABCD* gene cluster remained, containing the *pylA*, *gltS*, *lipA*, and *lipB* genes, which are typically proximal to the *csc* and *cssE* genes. Furthermore, 2 copies of the *rfbC*, *rfbA*, and *rfbB* gene cluster were identified in the NIID836 J_NmUC isolate; only 1 copy was found in NIID835 and NIID838 isolates. Although the *cps* locus in the 3 J_NmUC meningococcal strains were not identical to that in US_NmUC meningococci, the J_NmUC meningococci were genotypically nongroupable. All of the genetic features within the *cps* and *aniA-norB* loci confirmed that the 3 nongroupable ST11 J_NmUC meningococci were classified into the urethritis clade.

### *fHbp* Locus

In US_NmUC meningococci, fHbp was speculated to be highly expressed because the *fHbp* promoter sequence belonged to high fHbp–expressing promoter clade I (*33*). In the 3 ST11 J_NmUC *N. meningitidis* isolates, the *fHbp* promoter sequence, fHbp peptide, and *fHbp* allele were identical to those in US_NmUC meningococci strains (Appendix 2 Figure 2), suggesting fHbp might also be highly expressed in J_NmUC meningococci (*12*).

### Phylogenetic Analysis by Using WGS

**Figure 3:**
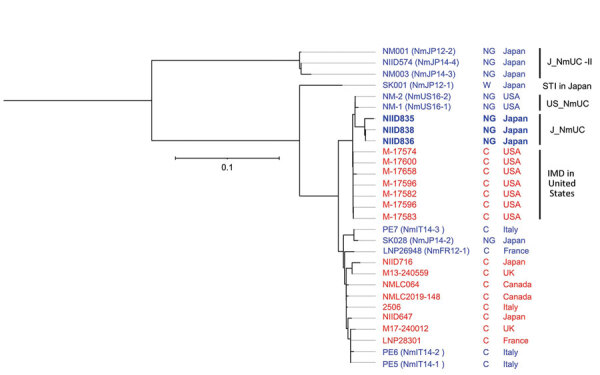
Phylogenetic analysis of *Neisseria meningitidis* from different countries in study of detection of novel US *N. meningitidis* urethritis clade subtypes in Japan. Strains isolated from patients with IMD (red font) or STI (blue font), serogroup (NG or C), and country of origin are indicated. US_NmUC, J_NmUC, and J_NmUC-II *N. meningitidis* isolates have detailed profiles (Appendix 1 Table). We included 1 sequence type 11 *N. meningitidis* strain isolated in Japan from a patient with an STI (SK028) and 4 serogroup C meningococci (MenC) that were phylogenetically close to SK028 (PE5, PE6, PE7, and LNP26948) (*29*). Moreover, we included 7 MenC phylogenetically close to US_NmUC (IMD strains in the United States) (*31*), 2 sequence type 11 MenC isolated from IMD patients during 2003–2020 in Japan (NIID647 and NIID716) (*28*), and 6 MenC phylogenetically close to the 2 MenC from Japan (*28*). Scale bar indicates nucleotide substitutions per site. C, serogroup C; IMD, invasive meningococcal disease; NG, nongroupable; STI, sexually transmitted infection.

To gain insights into the origin of J_NmUC meningococci, we performed phylogenetic analysis by using WGS to compare 9 ST11 IMD isolates from Japan (*29*), 1 STI isolate (NmJP12–1) (*30*), and 7 IMD MenC isolates from the United States that were genetically close to US_NmUC (*32*) (Figure 3). ST23 J_NmUC-II, ST11 serogroup W meningococci SK001 (NmJP12–1), 8 IMD MenC, and 5 STI MenC (*30*) isolates were genetically separate from J_NmUC and US_NmUC meningococci; 7 US IMD MenC that were close to US_NmUC (*32*) were also genetically close to J_NmUC. However, J_NmUC strains were the phylogenetically closest to US_NmUC, eliminating the possibility that J_NmUC was originally derived from MenC strains in Japan.

### Susceptibility to Antimicrobial Drugs

Antimicrobial resistance in *N. meningitidis* is considered to be acquired by transmission of genetic material from *N. gonorrhoeae,* such as the gonococcal *aniA-norB* locus (*14*). However, US_NmUC meningococci isolated in the United States were susceptible to the third-generation cephalosporin ceftriaxone, ciprofloxacin, and rifampin, whereas ≈75%–85% of US_NmUC meningococci were nonsusceptible (intermediate susceptibility) to penicillin G (*34*,*35*). The 3 J_NmUC meningococci were susceptible to most antimicrobial drugs tested, except the NIID836 strain had intermediate susceptibility to penicillin G, similar to US_NmUC meningococci (*34*,*35*). Those results suggest that genetic material related to antimicrobial resistance genes might not be transmitted into J_NmUC *N. meningitidis* isolates. Of note, the NIID835 strain was susceptible to penicillin G and ceftriaxone despite having the *penA327* allele, which typically reduces susceptibility to penicillin G and third-generation cephalosporins (*36*).

## Discussion

Meningococcus and gonococcus generally colonize distinct niches in humans causing systemic (meningococcus) and sexually transmitted (gonococcus) disease; few cases exist that identify *N*. *meningitidis* as a causative agent for STI (*14*). Meningococcal urethritis is symptomatically indistinguishable from gonococcal urethritis; one of the main problems in clinical and public health is that meningococcal urethritis cannot be diagnosed by the existing nucleic acid amplification test, a standard method for STI diagnosis (*14*,*34*). Urethritis clade meningococci, such as US_NmUC and J_NmUC, have been isolated only from urethritis patients (*10*), rectal swab samples of asymptomatic MSM (*18*), and 1 neonatal patient with conjunctivitis (*37*); virulence was considered equal to gonococci. However, urethritis clade meningococci were also speculated to colonize the upper respiratory tracts of sexual partners of persons who eventually manifested urethritis. No published studies exist regarding carriage of urethritis clade meningococci in the upper respiratory tract; thus, the public health threat of urethritis clade meningococci is unclear, and emergence of this clade should be continuously monitored. 

Although deletion of the *cps* locus or genes within this locus, which results in loss of encapsulation, is a main features of urethritis clade meningococci (*12*,*14*), the pattern of deletion within the *cps* locus was different between J_NmUC and US_NmUC isolates, despite the identical junctions between the *csc* gene and IS1301 sequences. Because meningococcal loss of encapsulation enhances adherence to human cells (*15*,*38*–*44*), loss of the capsule might promote *N. meningitidis*–induced urethritis. However, some cases of meningococcal urethritis might be caused by encapsulated *N. meningitidis* isolates (*30*). Therefore, the relationship between loss of encapsulation by deletions within the *cps* locus in *N. meningitidis* and meningococcal urethritis should be further examined.

Acquisition of the gonococcal *aniA-norB* locus (*12*) was another main feature of urethritis clade meningococci (Figure 1). In some *N. meningitidis* strains, such as M-17541 (Appendix 2 Figure 1), the meningococcal *aniA* gene was disrupted by an insertion or missense mutation (*45*,*46*). Moreover, if the meningococcal *aniA* gene was intact, expression was lower than that of gonococcal *aniA* genes (*45*). However, the gonococcal *aniA-norB* locus was not detected in some *N. meningitidis* isolates from patients with meningococcal urethritis (*30*), suggesting that acquisition of the gonococcal *aniA-norB* locus was advantageous (*12*,*13*,*17*) but not essential to cause urethritis.

A phylogenetic analysis using WGS data supports the hypothesis that US_NmUC and J_NmUC might be derived from the same ancestor (Figure 3). US_NmUC appears to have originated during 2006–2012 in the United States (*32*), and the ancestral strain might have been imported into Japan during the same period. However, MenC, serogroup W, and CC11 meningococci have rarely been detected in Japan for >40 years, even in IMD patients (*29*,*47*,*48*). Although CC11 meningococci have never been identified as a causative agent for meningococcal urethritis in Japan (*29*), J_NmUC meningococci, as well as the ST11 ancestral strain, might be dormant in the urethra or pharynx of persons in Japan. Therefore, further analyses of meningococcal isolates from healthy carriers and patients with urethritis will provide insights into dissemination of the *N. meningitidis* urethritis clade among the human population in Japan.

In conclusion, few studies have attempted to estimate the prevalence of meningococcal infections, including the urethritis clade. J_NmUC meningococci identified in this study are new subtypes of US_NmUC, and microbiologic characteristics, such as virulence and transmissibility, remain unclear. Continuous monitoring and analyses of J_NmUC meningococci will elucidate more precise features, including transmissibility and pathogenicity. Moreover, detection of J_NmUC in Japan suggests potential dissemination of several types of urethritis clade meningococci (US_NmUC and J_NmUC) worldwide. Global monitoring of urethritis-associated *N. meningitidis* isolates should be required to reveal further microbiologic and epidemiologic aspects of urethritis clade meningococci and to improve laboratory diagnostic testing for urethritis.

Appendix 1 Clinical and genetic isolate profiles for detection of novel US *Neisseria meningitidis* urethritis clade subtypes in Japan.

Appendix 2 Additional information for detection of novel US *Neisseria meningitidis* urethritis clade subtypes in Japan.
