# Supplementary material for: Detection of Novel US Neisseria meningitidis Urethritis Clade Subtypes in Japan
Source: Emerg Infect Dis. 2023 Nov;29(11):2210–7. doi: 10.3201/eid2911.231082 (PMC10617353; doi:10.3201/eid2911.231082)
Supplement: Appendix 2 — Additional information for detection of novel US Neisseria meningitidis urethritis clade subtypes in Japan. [file 23-1082-Techapp-s2.pdf]

Article DOI: https://doi.org/10.3201/eid2911.231082

EID cannot ensure accessibility for supplementary materials supplied by authors. Readers who have difficulty accessing supplementary content should contact the authors for assistance.

Detection of Novel US *Neisseria meningitidis* Urethritis Clade Subtypes in Japan

Appendix 2

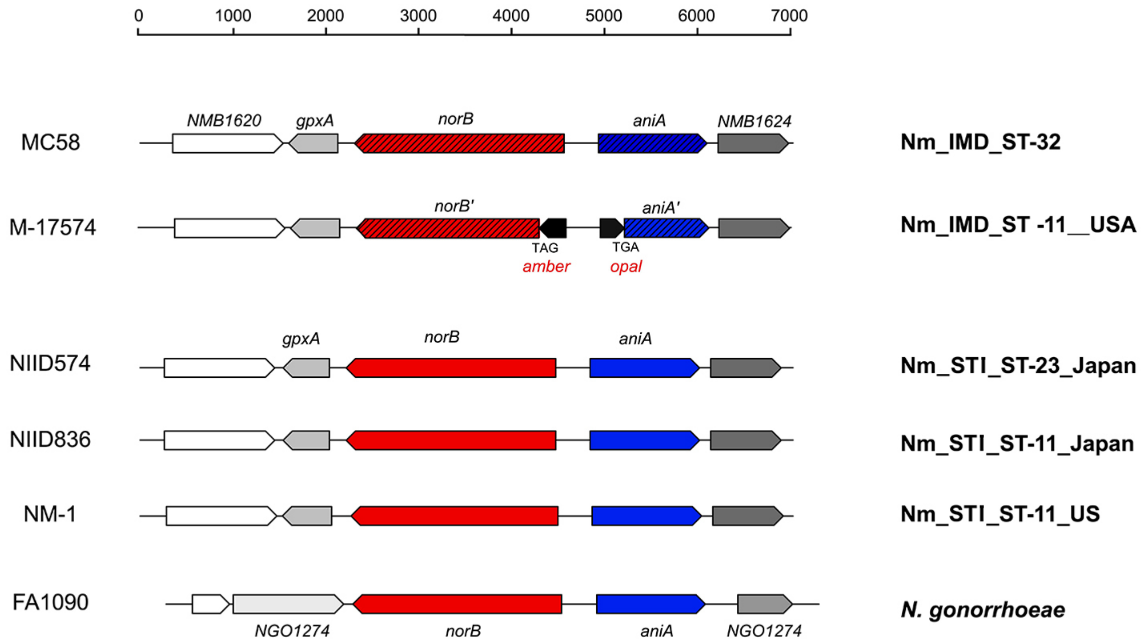

Appendix Figure 1. Gene alignments of the *aniA-norB* locus in *Neisseria meningitidis* and *N. gonorrhoeae* isolates. Strain names are on the left side. Descriptions on the right side are: Nm (*N. meningitidis*) or *N. gonorrhoeae*_ isolated from IMD patient (IMD) or from STI patient (STI)_sequence type (ST)_isolated in USA or Japan. The *norA* (red arrows) and *aniA* (blue arrows) genes are indicated. Open reading frames identical to NMB1620 (open white arrows) and NMB1624 (dark gray arrows) in MC58 are shown. IMD, invasive meningococcal disease.

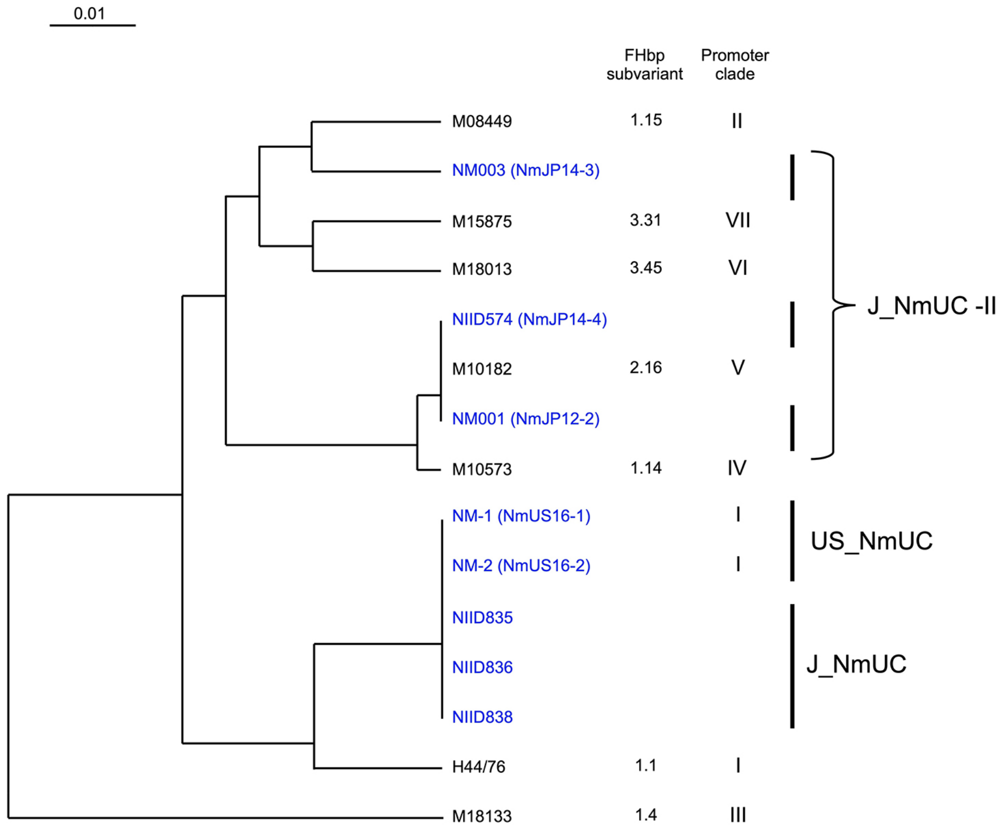

Appendix Figure 2. Phylogenetic analysis of the promoter region of the *fHbp* gene in different *Neisseria meningitidis* isolates from Japan and the United States. Strains were isolated from patients with urethritis (blue font) or patients with invasive meningococcal disease (black font). The fHbp protein subvariants and promoter clades were grouped according to the specific criteria (*32*, main text). Scale bar indicates nucleotide substitutions per site.
